# Self-care can be an alternative to expand access to universal health care: What policy makers, governments and implementers can consider for South Africa

**DOI:** 10.3389/frph.2022.1073246

**Published:** 2022-12-05

**Authors:** Athini Nyatela, Sizwe Nqakala, Leanne Singh, Taylor Johnson, Siphamandla Gumede

**Affiliations:** ^1^ Ezintsha, Faculty of Health Sciences, University of the Witwatersrand, Johannesburg, South Africa; ^2^ Department of Interdisciplinary Social Science, Utrecht University, Utrecht, The Netherlands

**Keywords:** personal health management, comprehensive care, linkage to care, equitable healthcare, HIV

## Abstract

As a result of collaboration amongst the various decision-makers in the field of healthcare, there has been an improvement in the access to healthcare and living conditions globally. Nonetheless, poorer communities continue to benefit the least from public investment. To bridge the gap, self-care can be a viable alternative as it allows individuals and communities to reduce their dependence on government healthcare solutions. Barriers to self-care do exist. Some of these are cost effectiveness, usability of self-care instruments, differentiated strategies and linkage to care. In identifying these obstacles, it is also worthwhile to investigate how they can be mitigated. To encourage sustained self-care in the HIV continuum, contextual factors as well as the manner in which individuals and communities engage with self-care must be considered. In South Africa, multiple variables come into play: literacy levels, cultural influences, socio-economic conditions and access to resources are some of these. Evidence demonstrates how self-care can be promoted by various stakeholders re-strategising to tweak and in some cases totally change existing structures. This paper explores some of the transformations, like at a governmental level where the supply of HIV self-testing kits is increased, at a production level where instructions for use are reformatting, in communities where sports programmes fulfil the dual purpose of developing sport skills and providing HIV education concurrently, and at an individual level where greater awareness invites greater participation in self-care. While self-care is a promising proposal, it is not a replacement for traditional health-care practices, but a complementary approach

## Introduction

The 1978 Alma-Ata declaration (1) directs governments and all stakeholders within the primary healthcare domain to promote and facilitate equitable healthcare for all. Some of the commendable global milestones achieved as a result, are that childhood deaths have decreased significantly, availing essential drugs has become more commonplace and antenatal care has improved (2). Unfortunately, the financially privileged continue to benefit more from public/government investment thus consuming the most care, at the expense of the poor, for whom the opposite holds true (3, 4). The global response to the COVID-19 pandemic exemplifies this unfortunate reality where inequity with regard to COVID-19 vaccine production and access persists (5). Given the impact that a country's economic state has on the quality of primary healthcare it provides, it would seem prudent to explore alternatives than depending solely on governments to satisfy citizens’ healthcare needs. Self-care is one such alternative.

The WHO defines self-care as “the ability of individuals, families and communities to promote health, prevent disease, maintain health, and to cope with illness and disability with or without the support of a healthcare provider” (6). Working within the ambit of this definition, self-care will primarily refer to activities related to the self-management of health, such as: lifestyle modifications and behaviour change, self-screening and self-testing (e.g., for HIV and Hepatitis), self-monitoring (e.g., tracking blood sugar and blood pressure levels), self-management of acute illnesses and adherence to medication. With the rapid increase in self-care interventions, there is a shift in the way healthcare is perceived, understood, and accessed (7).

In South Africa, the need for self-care is becoming increasingly apparent. Patients seeking care have frequently not been able to receive it due to service-delivery shortfalls (8, 9). This, coupled with the fact that by 2019, 84% of South Africans depended on public health systems to fulfil their healthcare needs (8). Self-care interventions can unburden primary healthcare clinics (PHCs), and provide convenient ways for patients to manage their health. During the Covid-19 pandemic, the need for self-care interventions became more evident particularly for patients with co-morbidities like diabetes mellitus, since self-care can allow diabetes patients to engage in healthcare behaviour that makes them more likely to survive Covid-19, should they contract it (10). For self-care to be feasible as an alternate means of accessing health care and decongesting facilities, cost effectiveness, usability of self-care instruments, differentiated self-care strategies and linkage to care concerns need to be considered.

**Figure 1:**
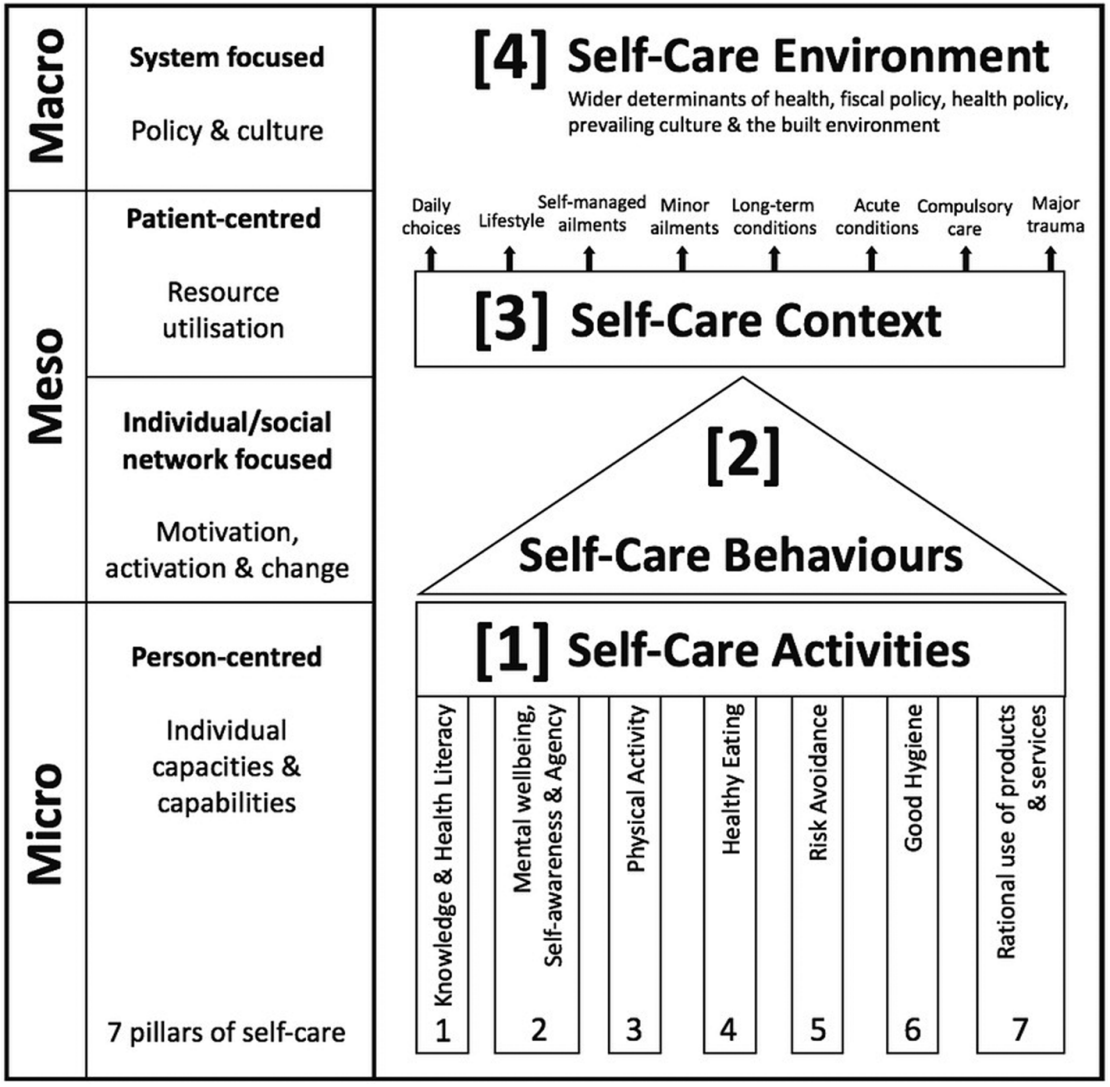
The self-care matrix (SCM).

In South Africa, healthcare coverage particularly in relation to HIV and Tuberculosis declined in some regions due to COVID care taking precedence over routine health services (11). In order to mitigate for this and losses resulting from other possible unforeseen future health crises, self-care may well be a practical solution. While this may be so, El-Osta A, et al. in their self-care matrix (Figure 1) illustrates that self-care does not begin and end with the patient. For it to be workable and sustainable, it needs the support of both community (at a meso level) and policy-makers (at a macro level). For example, while an HIV positive patient might adopt a healthier life-style, they still require support within their communities by being able to easily access the assistance of health care workers in their vicinity, but this can only be possible if systems and policies are in place to supply the required number of HCW and provide the necessary facilities (12).

### Viability of self-care

#### Cost effectiveness

While the functional approach to increasing access to healthcare is to reduce its cost, it should not compromise the quality of healthcare (13). With regard to self-care, the financial demands it places on the patient affects the extent to which they can interact with self-care activities, irrespective of the patient's desire to embrace it (14).

Because of the nature of self-care interventions like self-monitoring, or self-screening, people expect such interventions to provide them with immediate or direct benefits (15). However, these benefits may not outweigh their costs, particularly if individuals are responsible for the costs themselves. Although self-care often means self-financed, ideally, interventions begun in health facilities and shifted to home settings should not garner additional costs to the patient nor require further subsidisation. There is a fair amount of evidence to suggest that self-care could reduce direct patient costs and the risk of financial hardships (16). The HIV Self-Testing in Africa (STAR) Phase 2 Evaluation Report shows how HIV self-testing (HIVST) cost will potentially decrease as newer products enter the market (17).

Furthermore, patients who opt for self-care, can often obviate their transport costs and needing to visit hospitals for HIV related care (18). This is crucial for patients in low income settings whose financial constraints are a barrier to seeking medical attention (19). Cost efficiency resulting from self-care can extend beyond the patient too. Increased numbers of patients who engage in self-care practices, means that healthcare facilities and healthcare workers will likely experience reduced and possibly manageably patient loads (20). Thus, health systems stand a greater chance of being more efficient and more targeted (16).

Various reports estimate large cost savings from self-care, however, whether all patients are able to care for themselves competently independently of a healthcare worker must be considered.

#### Usability of self-care instruments

Self-care interventions play a pivotal role in the prevention, identification, management, treatment optimisation, and decreasing disease incidence (21). For self-care to be a viable option, patients need to demonstrate a level of self-efficacy, which can be defined as a person's belief in their own ability to organise their behaviour for the purpose of achieving the desired outcome (22). Within the healthcare context, this would mean patients being able to engage with instructions for use (IFU), instruments and equipment and availing alternate monitoring methods in an independent manner.

Independence hinges on the end user being able to comprehend instructions for the use of medication and instruments (23). In South Africa, over 4 million adults are illiterate (24, 25), so it is to be expected that for poorly literate patients, there will be limitations in their ability, particularly when it comes to comprehending written IFU, resulting in the correct engagement with various self-care activities being compromised. One common effect of poor comprehension of IFU is patients taking the incorrect dosage of medication (26, 27).

To combat the pre-requisite of being literate for a person to be able to adopt self-care, methods of information dissemination that go beyond the written word, such as videos, need to be employed (28, 29). Similar considerations need to be made with regards to the language used in IFU, given that SA has 11 official languages. Therefore, for successful use of self-monitoring devices, patient autonomy and its relationship to literacy and comprehension levels of end-users must be considered (30).

Compared to people without HIV, people living with HIV (PLHIV) find themselves twice as likely to be at risk for cardiovascular disease (CVD) and their mortality risk increases if the CVD risk is left unmitigated (31). Hypertension (HTN) is a recognised contributor to PLHIV developing CVD, yet its monitoring particularly in Sub Saharan Africa remains underwhelming. Among the many reasons for this, like stigmatisation and insufficient personnel, non-optimisation of blood pressure (BP) monitoring devices is one (32). Teshome DF et al. identify the lack of sufficient BP monitors and appropriately skilled health extension workers as being hindrances to home-based HTN screening in a rural Ethiopian region. They put forward that establishing systems that address the minimising of these obstacles would render home-based HTN monitoring to be a practicable option (33). Moreover, availing testing instruments and equipment, like blood pressure (BP) monitors and wearables to patients, which can be used in their homes, is a further means to promote independence. Such is the need that the WHO developed interventions for primary health care in low-resource settings, some of which include the self-monitoring of BP to manage hypertension for example, where access and cost are not hindering factors (14).

On a systemic level, digital health platforms provide further solutions to promote self-care, as these also have the advantage of allowing patients to monitor (34) their health without needing to visit healthcare facilities. South Africa has a significant rural population for whom accessing healthcare services is often problematic. Rural communities can benefit significantly from telehealth offerings that would otherwise be unavailable to them in person (35). This possibility becomes particularly important when information needs to be disseminated urgently, as we experienced during the Covid-19 pandemic. Research into the viability of HIV related health apps like iThemba (36) which facilitate the monitoring and medication of HIV patients, have already shown that they can be beneficial to patients who ordinarily would need to visit clinics at short intervals and pick up medications at centralised healthcare facilities.

Irrespective of how encouraging these interventions are, overcoming the current obstacles to its uptake needs more robust efforts to mitigate the costs and practicalities associated with establishing e-/ telehealth pathways (37, 38). A further complement to patients being able to monitor their own health, is the strengthening of individual and community engagement in the promotion of healthcare.

#### Differentiated healthcare

Among the variables which influence an individual's willingness to embrace self-management of their health, are the intersectionalities of culture, sex, gender, age and class (39, 40). To address particular needs of various sections of society, differentiation in health-management is a practical approach. Differentiated care support is not new in South Africa's healthcare system, and was envisioned as a client-centred approach that simplifies and adapts chronic services to manage HIV across the cascade of care, while decongesting burdened health facilities (41).

Differentiated care support needs to expand, to address the additional self-care activities required for effective self-management of chronic conditions. Some of these additional interventions include education and community engagement to promote behaviour and lifestyle modifications (42).

Health education is pivotal in promoting healthy life-styles, encouraging individuals to take accountability for their health (43). Perhaps to kickstart community-based initiatives, investing in national educational programmes that target youth might be a feasible approach. The Grassroots SS (44) of which SKILLZ Street (45) is an example of how health education can be included within youth empowerment projects. In this initiative, young girls learn about their sexual reproductive health and rights, and general life skills which are essential in steering their decision making in terms of sexual behaviour and reproductive health (44–46). While innovative educational strategies such as this are promising, such interventions need to be enhanced and sustained as innovative health education strategies have been shown to enjoy success when entrenched at a structural level (29, 47).

However, society does not only have to rely solely on directives from health authorities in how to manage their health. Malama K et al. (2022) explain that ensuring that communities have adequate education on HIV self-care practices as well how to avail such, can play a significant role in promoting HIV self-care (48). This is especially true for vulnerable populations like the elderly. A recent South African study revealed that healthcare for the aged particularly in low-resource communities is sorely lacking in patient-centredness, resulting in inadequate health care for this sector of the population (49). A means to overcoming this marginalisation of the elderly (43) is to provide them with the skills and opportunities that they require to adopt and maintain self-care whenever possible. Studies conducted on the viability of self-care among older persons following an acute illness admission show a decline in ones' s ability to perform activities of daily living such as bathing, eating, and walking (50, 51), therefore, one's ability to self-care post hospitalization necessitates binary support systems such as home based care (52).

Knight L, et al. (2018) suggest the formulation of *Chronic Care Clubs* in a community setting, which decentralise HIV treatment and monitoring for the elderly with co-morbidities, thereby providing them with a workable way to manage their conditions (53). Understanding these dynamics, and their implications can provide valuable insight to allow for the planning and execution of alternative healthcare interventions to cater particularly to people in low income and vulnerable communities (54), including women who require emergency maternal care (55).

On a more individualised level, differentiation can also take into consideration the cultural (40) values and attitudes that influence gender roles. In African and Asian societies, these values play a major role in health decisions: men are largely the sole decision makers in their families. However, studies show that in such circumstances, women are often eager to bypass the cultural expectations. For example, when offered self-care approaches to HIV testing, most women in rural SA opted for HIV self-testing because they felt more empowered, and independent (56).

Thus it is important to understand all enabling factors that help or disturb self-care behaviours and strategize ways in which to mitigate them. In doing so, deep-rooted behaviour and attitudes which are detrimental to health and self-care may be able to be changed.

Policy makers, implementers and governments should also consider fostering collective healthy living through community exercise sessions (42), to support self-care practice.

#### Delayed linkage to care concerns

Linkage to care is a self-management intervention which offers strategies that promote active participation of individuals in their health, and ways to reach improved outcomes like increased access to testing (57). However, for self-care to be viable, patients must understand that synergies between self-care, and facility-based health provision are negotiated by the severity of a condition, the complexity of its care, and the expertise required to manage a patient's health (13). So, despite the promising future of self-care, it must be remembered that practising self-care activities is an accompaniment to, not an obliteration of the need to link to professional care.

The term “linkage to care” was first coined to define a patient's initial clinical visit, following an HIV positive, and most recently, reactive self-screening result. It is regarded as a crucial step in the management of HIV and viral suppression (58). In South Africa, while HIV self-screening can be attributed to an increase in access to HIV testing, it is also reported to have worsened the rate at which people link to care (59).

Evidence points to financial constraints and time needed to access health facilities, patient and provider relationships (60–62), as well as transport vulnerability (63, 55) as being key indicators to delayed care. Like in Kenya and Tanzania, decreased linkage and retention in care in SA can also be attributed to discrimination (64) and staff attitudes (65, 66).

While various initiatives such as mobile health clinics (67) for hard to reach communities, mobile health for pregnant mothers (68) and Pulmonary Tuberculosis patients (69),, self-triage apps for acute illnesses (70) and community tracking initiatives have been instrumental in addressing some of the gaps in linkage to care, a lot of work is required in addressing barriers to facility attendance, such as patient satisfaction and provider communication (71).

Limited access to the internet, language barrier and in some instances, infrastructural resources such as mobile health cars can pose limitations to these recommendations, particularly in poorer countries.

## Discussion

The integral role that self-care plays in empowering lay people to assume responsibility for their own health has been advocated as a method to mitigate non-communicable diseases (NCD) (72) and most recently, even the COVID-19 pandemic (73).These interventions necessitate varying levels of self-care activities on the part of the patient, and to be successful, patients must adhere to medication and lifestyle modifications, and with HIV, self-monitor their conditions. Some factors which need to be considered when contemplating the extent to which self-care can be initiated and sustained is cost effectiveness, usability of self-care instruments, differentiated self-care strategies and linkage to care.

A key driver in self-care decision making is the financial implications at personal and institutional levels. There is an acknowledgement that access to health insurance plans have a positive impact on a patient's inclination to enter into self-care (74). For self-care interventions to be sustainably financed, a combination of government subsidies, private financing, insurance coverage, and partial out-of-pocket payments will need to be considered. In some countries with social health insurance, insurance providers partially cover healthcare, and various studies/reports estimate large cost savings from self-care.. Be that as it may, the health system is still accountable for the outcomes from the use of self-care and needs to closely monitor the economic consequences of self-care (16, 21).

Apart from economic concerns, in South Africa for example, one of the barriers to linkage to care in HIV patients is that while patients are willing and able to collect medication from alternative facilities like community based ones, the lack of interface with health workers resulted in patients being more reluctant to visit these facilities (75). The fear of stigmatisation, real or perceived, around having HIV is also a considerable barrier to care linkage (76). A recent study highlights how urban and rural communities approach self-management of HIV differently, where rural communities are less keen to embrace support because of fears of being discriminated against (77). In light of the above-mentioned concerns, it is clear that even though efforts can be made to reduce the costs involved with linking to care, more effort needs to be placed in mitigating the personal distress of HIV patients, which are often borne from cultural and context-related biases.

Creating awareness related to one's health is an integral part of self-care, and one study postulates that awareness of the indications of having contracted HIV plays the most powerful role in whether an individual links to care (78). In response to the COVID-19 pandemic, coproduction has been suggested as a means for communities to minimising their reliance on government-initiated healthcare strategies. Furthermore, communities can also draw on their own experiences and strategise ways in which to work around the barriers that compromise their engagement in personal health maintenance (79). The same argument could well be applied to motivating communities to extend responsibility for their health beyond COVID-19.

While the self-care interventions discussed in this paper are drawn from the lessons learned from HIV and/or COVID-19, the paper points out common issues about self-care that are relevant to other health conditions, such as Diabetes (54).

Self-care interventions will not work in isolation and should not replace traditional, conventional healthcare. Further research is required to understand how best to link self-care practices to service delivery in facilities. Therefore, to bridge the gap between self-management of disease, treatment, and support, policy makers and governments should consider how linkage to care can be facilitated.

## Conclusion

To reimagine health systems that can withstand pressure from growing health concerns, a portion of healthcare must be patient-led, and self-administered. However, while self-care interventions are an important aspect of healthcare provision, and can address some barriers, such as waiting times, privacy, confidentiality and cost, self-care practices do not negate the need to visit a health facility for further management.

## Data Availability

The original contributions presented in the study are included in the article/Supplementary Materials, further inquiries can be directed to the corresponding author/s.
